# A Rare Case of Uterine Artery Pseudoaneurysm After Hysteroscopic Management of Trophoblastic Disease

**DOI:** 10.1155/crog/6367800

**Published:** 2026-05-30

**Authors:** Lela Tandashvili, George Jinchveladze, Lela Iremadze, Sofio Skliarenko, Irina Gagua

**Affiliations:** ^1^ Department of Obstetrics and Gynecology, Georgian American University, Tbilisi, Georgia; ^2^ Department of Obstetrics and Gynecology, Aversi Clinic, Tbilisi, Georgia; ^3^ Department of Vascular Surgery, Caucasus Medical Center, Tbilisi, Georgia; ^4^ Department of Medicine, The University of Georgia, Tbilisi, Georgia, uga.edu; ^5^ Department of Oncology, Ivane Javakhishvili Tbilisi State University, Tbilisi, Georgia, tsu.ge

**Keywords:** computed tomography angiography, delayed postpartum hemorrhage, hysteroscopy, transcatheter embolization, uterine artery pseudoaneurysm

## Abstract

**Introduction:**

Uterine artery pseudoaneurysm (UAP) is a rare but potentially life‐threatening condition, most commonly associated with childbirth and less frequently with intrauterine procedures such as dilatation and curettage or hysteroscopy. Its incidence is estimated at 3–6 per 1000 deliveries, whereas occurrence in the context of gestational trophoblastic disease (GTD) is extremely uncommon. UAP may remain asymptomatic before presenting as sudden vaginal hemorrhage days to weeks after uterine intervention. Prompt recognition and timely imaging are essential for effective management. Computed tomography (CT) angiography is considered the gold standard, whereas Doppler ultrasound and magnetic resonance imaging (MRI) are valuable alternatives. Uterine artery embolization is the preferred treatment due to its high success rate and fertility‐preserving potential.

**Case Presentation:**

We report an exceptionally rare case of UAP developing 5 weeks after hysteroscopic removal of retained products of conception in the context of GTD. Power Doppler ultrasonography demonstrated a hypervascular lesion with a characteristic swirling flow pattern, confirmed by contrast‐enhanced CT. The patient underwent successful selective uterine artery embolization, resulting in complete symptom resolution. Over 5 years of follow‐up, she remained recurrence‐free, with regular menstruation and a subsequent full‐term delivery, indicating preserved reproductive function.

**Discussion:**

This case highlights the importance of considering UAP in delayed posthysteroscopic hemorrhage, particularly in GTD. Multimodal imaging is essential for accurate diagnosis and differentiation from other vascular lesions. Selective uterine artery embolization is a safe, effective, and fertility‐preserving treatment with favorable long‐term outcomes, including successful pregnancy.

## 1. Introduction

Uterine artery pseudoaneurysm is a rare but potentially life‐threatening vascular lesion that may occur after uterine trauma, surgical procedures, or childbirth. It often remains clinically silent until sudden hemorrhage develops days to weeks following interventions such as hysteroscopy, dilatation and curettage, or cesarean delivery [1–3].

UAP represents a contained arterial rupture with persistent communication to the arterial lumen, leading to sudden or intermittent vaginal bleeding. Unlike the diffuse bleeding seen in GTD, UAP typically causes episodic, arterial hemorrhage. Although UAP may mimic uterine arteriovenous malformations (AVMs), accurate differentiation is essential because management strategies differ and delayed diagnosis may result in severe bleeding [4].

The incidence of UAP is estimated at 3–6 per 1000 deliveries [5], whereas its occurrence in the context of GTD is extremely uncommon and likely underreported due to diagnostic challenges [2, 3, 6].

Diagnosis relies on imaging. Power Doppler ultrasound typically demonstrates a discrete lesion with a characteristic “yin–yang” flow pattern [7, 8], whereas AVMs present as multiple tortuous high‐flow vessels [9, 10]. CT angiography and MRI assist in anatomical evaluation, whereas digital subtraction angiography (DSA) provides definitive diagnosis and therapeutic access [4, 11, 12].

Selective uterine artery embolization is the preferred treatment, offering effective hemostasis while preserving fertility [13]. However, long‐term outcomes, particularly regarding future pregnancies, remain limited.

We report an extremely uncommon case of UAP following hysteroscopic removal of retained products of conception in the context of GTD. The patient was successfully treated with selective uterine artery embolization and subsequently delivered at term, demonstrating preserved reproductive function. This case highlights the importance of early recognition, accurate imaging, minimally invasive management, and provides rare long‐term evidence supporting fertility preservation after treatment.

## 2. Case Report

**Figure 1:**
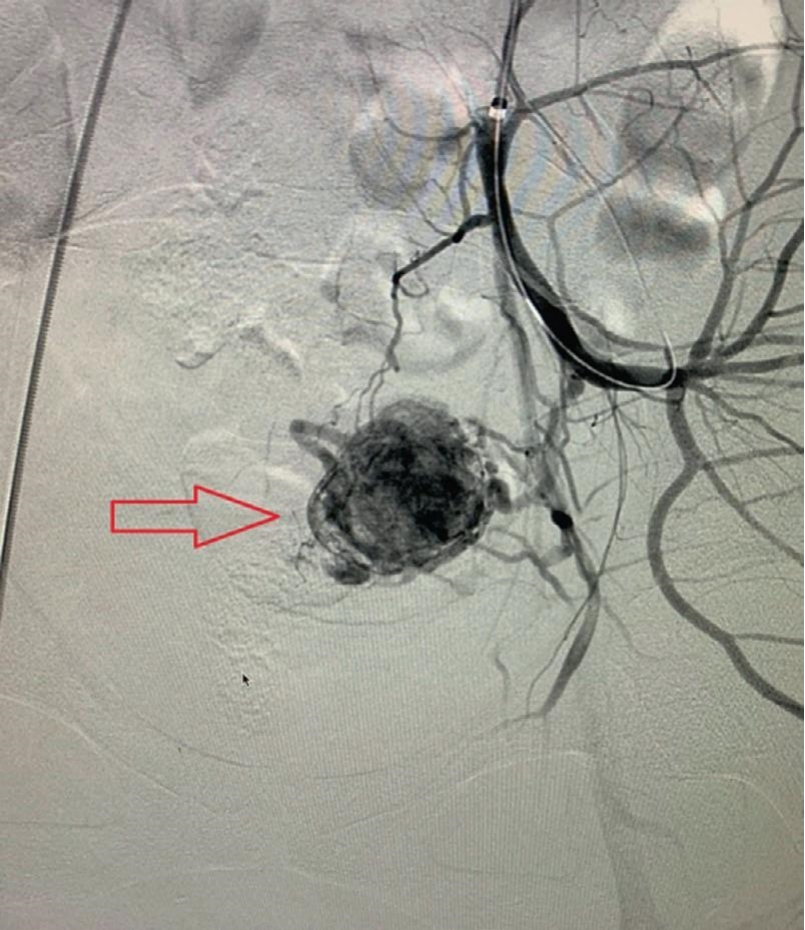
Digital subtraction angiography (DSA) demonstrating a contrast‐filled pseudoaneurysm arising from a branch of the uterine artery (indicated by arrow). The image reveals an abnormal vascular collection with delayed contrast pooling, consistent with a uterine artery pseudoaneurysm. This angiographic finding confirmed the source of ongoing delayed uterine bleeding and guided subsequent embolization therapy.

**Figure 2:**
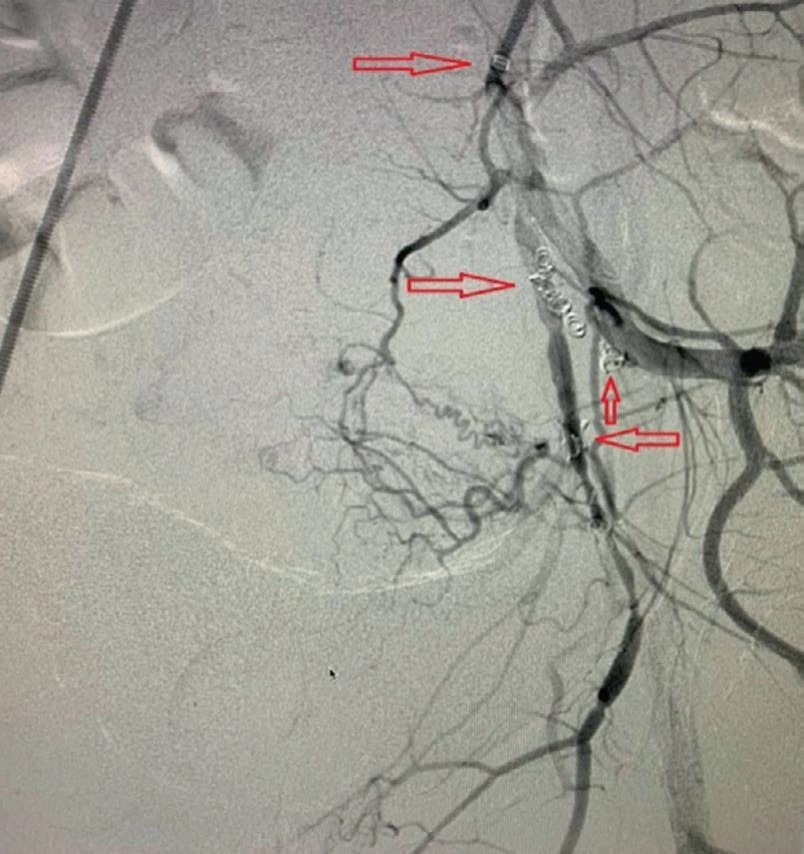
Postembolization digital subtraction angiography showing successful occlusion of the left uterine artery with no evidence of abnormal vascular flow. The image confirms effective hemostasis and resolution of abnormal vascular flow.

A 33‐year‐old woman presented to the emergency department with acute vaginal bleeding and weakness. She appeared mildly pale but was hemodynamically stable (blood pressure 100/70 mmHg, heart rate 98 bpm, respiratory rate 17/min). On pelvic examination, active vaginal bleeding was observed without a clearly identifiable cervical or vaginal source, suggesting a uterine origin. One and a half months earlier, she underwent hysteroscopic removal of retained products of conception in the setting of gestational trophoblastic disease, with histopathology confirming the diagnosis. Since then, she had experienced intermittent heavy bleeding with clots. Her obstetric history included one cesarean delivery 5 years earlier. Laboratory tests showed hemoglobin 9.5 g/dL (reference 12–15.5), with normal platelets and coagulation. Power Doppler ultrasonography demonstrated a 30 × 28 × 29 − mm hypoechoic lesion in the anterior uterine wall with the characteristic “yin–yang” sign. CT angiography confirmed UAP. Serum *β*‐hCG was 1690 mIU/mL (reference < 5), consistent with gestational trophoblastic disease. Given these findings, DSA was performed, revealing a pseudoaneurysm from a branch of the left uterine artery (Figure 1). Selective embolization was carried out via left brachial artery access using the Seldinger technique. Postembolization DSA showed complete exclusion of the lesion (Figure 2). The patient tolerated the procedure well and was discharged 2 days later. *β*‐hCG levels declined to < 100 mIU/mL over the following weeks.

**Figure 3:**
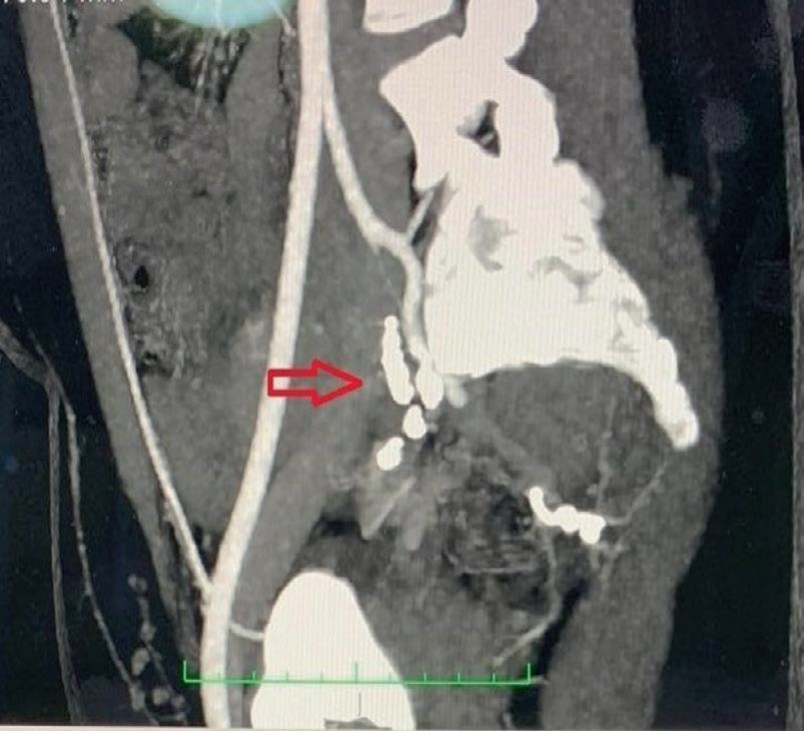
Follow‐up contrast‐enhanced computed tomography (CT) imaging performed at 1‐month postselective uterine artery embolization demonstrates stable postprocedural findings. Sagittal and coronal reformatted views reveal the presence of embolization coils at the previously treated site of the uterine artery pseudoaneurysm (denoted by red arrows). There is no evidence of active contrast extravasation, residual perfusion, or recurrence of the pseudoaneurysm.

At 1‐month follow‐up, she was symptom‐free, and imaging confirmed no recurrence (Figure 3). She was advised close monitoring in future pregnancies. Over 5 years of follow‐up, she remained recurrence‐free, menstruated regularly, and delivered a healthy infant.

Given the single‐patient design of this case report, no formal statistical analyses were conducted; all findings are presented descriptively. This case report was prepared in accordance with the CARE guidelines.

Written informed consent was obtained from the patient for publication of this case report and any accompanying images.

## 3. Discussion

This case highlights the importance of considering uterine artery pseudoaneurysm in the differential of delayed postprocedural bleeding, even after minimally invasive procedures. Early recognition and intervention prevent catastrophic hemorrhage.

Accurate diagnosis relies on multimodal imaging. Doppler ultrasonography is typically first‐line, whereas CTA and DSA provide definitive diagnosis and enable therapeutic planning. Differentiation from other vascular lesions, including AVMs, is crucial, as management differs.

Selective uterine artery embolization is the preferred treatment due to its minimally invasive nature, high success rate, and fertility‐preserving potential.

Despite the presence of GTD, clinical and imaging findings indicate that the UAP was the primary source of hemorrhage. Bleeding was sudden and episodic, imaging demonstrated a discrete pseudoaneurysm with the characteristic “yin–yang” flow, and selective embolization immediately controlled the bleeding, confirming the diagnosis. The uterine artery embolization was performed exclusively for hemostasis and was not intended as treatment for the underlying gestational trophoblastic disease.

This case is notable for UAP in the context of GTD, a rarely reported association. Favorable long‐term outcomes—including 5‐year recurrence‐free, normal menstruation, and a subsequent term delivery—demonstrate embolization′s safety and fertility‐preserving efficacy. This report provides rare long‐term evidence and reinforces the importance of prompt diagnosis and minimally invasive UAP management.

## Author Contributions

The authors take full responsibility for this article.

## Funding

No funding was received for this manuscript.

## Disclosure

A preprint version of this manuscript was previously published on Zenodo in 2025 by Lela Tandashvili (corresponding author), George Jinchveladze, Lela Iremadze, Sofio Skliarenko, and Irina Gagua. All authors have read and approved the final version of the manuscript. Lela Tandashvili had full access to all of the data in this study and takes complete responsibility for the integrity of the data and the accuracy of the data analysis. Lela Tandashvili affirms that this manuscript is an honest, accurate, and transparent account of the study being reported; that no important aspects of the study have been omitted; and that any discrepancies from the study as planned (and, if relevant, registered) have been explained.

## Ethics Statement

The authors have nothing to report.

## Consent

Written informed consent was obtained from the patient for publication of this case report and any accompanying images.

## Conflicts of Interest

The authors declare no conflicts of interest.

## Data Availability

The authors confirm that the data supporting the findings of this study are available within the article.
